# Survival trends of gastrointestinal stromal tumor in real-world settings: a population-based retrospective study

**DOI:** 10.3389/pore.2025.1611896

**Published:** 2025-03-04

**Authors:** Guohua Jia, Xiangpan Li

**Affiliations:** ^1^ Cancer Center, Renmin Hospital of Wuhan University, Wuhan, Hubei, China; ^2^ Department of Oncology, Renmin Hospital of Wuhan University, Wuhan, Hubei, China

**Keywords:** GIST, survival, tumor-specific mortality, trend, outcome

## Abstract

**Purpose:**

This study aims to evaluate whether survival outcomes for GIST patients have improved over the past decades and to identify the specific patient subgroups that have benefited from advances in treatment.

**Patients and methods:**

A total of 4,127 GIST patients diagnosed between January 1980, and December 2019, were included in this study using data from the Surveillance, Epidemiology, and End Results (SEER)-9 Registries. Survival differences among GIST patients were analyzed across five time periods (1980–1999, 2000–2004, 2005–2009, 2010–2014, and 2015–2019) and within demographic, neoplastic, temporal, economic, and geographic categories using the log-rank test. Multivariable Cox regression models were employed to identify risk factors associated with GIST-specific survival. Associations between time periods and GIST-specific mortality (TSM) were examined using a multivariable Cox regression model.

**Results:**

Survival outcomes for GIST patients significantly improved in the 2000–2009 period but showed no substantial improvement in the 2010–2019 period. After adjusting for age, gender, tumor location, ethnicity, tumor stage, median household income, and geographic area, the multivariable Cox regression models revealed that older age (≥65 years) (HR = 1.977, 95% CI = 1.470–2.657), tumors located outside the gastrointestinal tract (HR = 1.505, 95% CI = 1.267–1.786), regional lesions (HR = 2.225, 95% CI = 1.828–2.708), and distant lesions (HR = 5.177, 95% CI = 4.417–6.069) were independent risk factors for TSM (p < 0.05). After adjusting for time periods and age, gender, tumor location, tumor stage, median household income, patients in 2000–2004 (HR = 0.662, 95% CI = 0.523–0.839), 2005–2009 (HR = 0.431, 95% CI = 0.339–0.549), 2010–2014 (HR = 0.437, 95% CI = 0.341–0.561), and 2015–2019 (HR = 0.365, 95% CI = 0.273–0.489) had a significantly lower risk of TSM than patients in 1980–1999 (p < 0.05). Similarly, patients in 2005–2009 (HR = 0.661, 95% CI = 0.555–0.788), 2010–2014 (HR = 0.696, 95% CI = 0.578–0.838), and 2015–2019 (HR = 0.607, 95% CI = 0.476–0.773) also had a significantly lower risk of TSM than patients in 2000–2004 (p < 0.05). However, patients in 2010–2014 (HR = 1.042, 5% CI = 0.863–1.258) and 2015–2019 (HR = 0.945, 95% CI = 0.734–1.216) did not have a significantly lower risk of TSM compared to patients in 2005–2009 (p > 0.05).

**Conclusion:**

GIST survival has significantly improved during the period 2000–2009 but showed no substantial improvement in 2010–2019, with the turning point for lower risk of TSM being 2005. Innovative strategies are needed to further improve survival outcomes for GIST patients, particularly for older patients and those with tumors originating outside the gastrointestinal tract.

## Introduction

Gastrointestinal stromal tumors (GISTs) are the most common subtype of sarcoma, with an incidence of approximately 1.2 cases per 100,000 individuals per year [1]. GISTs primarily occur in the stomach, small intestine, colorectum, and, less commonly, in other locations outside the gastrointestinal tract. GISTs arising from different primary tumor sites exhibit distinct clinical features and outcomes [2, 3].

Complete surgical resection remains the curative option for localized GISTs. Since GISTs are generally resistant to chemotherapy and radiation, patients with inoperable advanced GISTs have historically faced a poor prognosis, with a median overall survival of approximately 18 months before recent therapeutic advances [1]. Over the past few decades, the widespread adoption of routine physical examinations has enabled the early detection of cancers, and the development of novel antitumor therapies, including targeted drugs and immunotherapy, has significantly improved the prognosis for patients with cancers such as lung, liver, and breast cancer [4–6]. However, there is a limited amount of research that has addressed whether GIST patients have similarly benefited from advances in diagnostic and therapeutic strategies.

In addition, with the introduction of new antitumor drugs, the prognosis for GIST patients may have evolved. For instance, Jason S. Gold [7] reported that gastric GISTs were associated with better survival outcomes compared to small bowel GISTs, whereas Ulrich Guller [8] presented conflicting findings. These discrepancies are likely due to differences in the periods during which study subjects were enrolled. Therefore, it is crucial to determine when GIST patients have experienced improved prognosis due to advances in antitumor therapies.

Moreover, GIST is a heterogeneous disease, with outcomes influenced by factors such as primary tumor site, age, gender, and disease stage. It is essential to identify the specific subgroups of GIST patients who have benefited from modern diagnostic and therapeutic measures [9]. Insights from this large-scale, retrospective cohort study will provide up-to-date epidemiological knowledge on GISTs. They will aid in identifying targeted patient populations to further improve survival outcomes in the future.

## Materials and methods

### Population database

The data utilized in this study were obtained from the Surveillance, Epidemiology, and End Results (SEER) program of the U.S. National Cancer Institute^1^. The SEER database contains population-based cancer registry data that provide comprehensive information on cancer patients, such as demographic characteristics, neoplastic features, and survival outcomes. Access to the SEER database was granted following the completion of a data use agreement.

**FIGURE 1:**
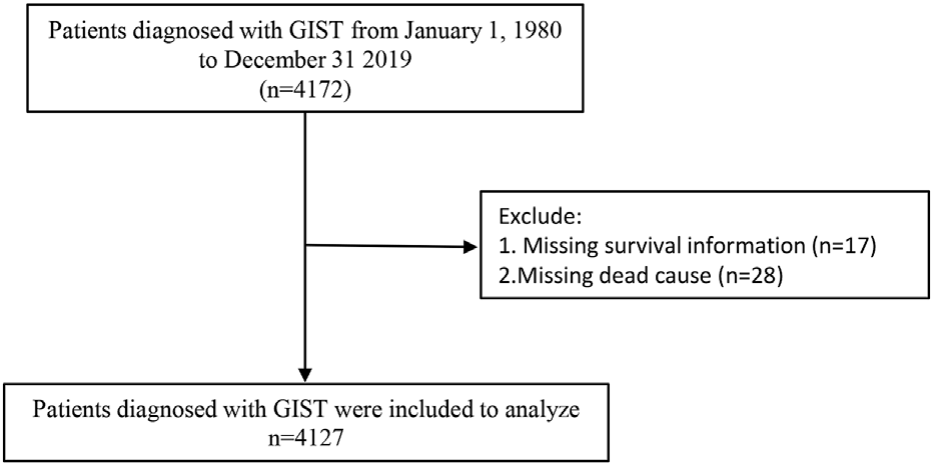
Flowchart of patient selection. Notes: Abbreviations: GIST: Gastrointestinal Stromal Tumor.

To analyze trends in GIST over the longest possible time period, we utilized the SEER-9 registries program. These nine registries cover the San Francisco-Oakland SMSA, Connecticut, Detroit (Metropolitan), Hawaii, Iowa, New Mexico, Seattle (Puget Sound), Utah, and Atlanta (Metropolitan). The data used in this study were derived from the SEER Research Data, 9 Registries, November 2021 Submission (1975–2019) and downloaded using SEER*Stat software (version 8.3.9.2, NY, United States). GIST patients were included in the study cohort based on the following criteria: 1. Tumor histology is classified as GIST according to the ICD-O-3 code. 2. Malignant behavior. Patients were excluded if they had missing data on survival status (17 patients) or unknown cause of death (28 patients). After exclusions, a total of 4,127 GIST patients were included in the final study cohort. A detailed flowchart illustrating the patient selection process is shown in Figure 1.

### Parameter definition

All information (age, gender, ethnicity, primary tumor site, disease stage, year of diagnosis, economic status, and geographic data) of GIST patients was obtained from the previously described SEER database. Age was categorized into the following groups: young adults (0–39 years), middle-aged adults (40–64 years), and older adults (≥65 years). Subjects were classified as white or non-white based on ethnicity codes. Primary tumor sites were divided into four categories: (1) stomach, (2) small intestine, (3) colorectum, and (4) outside the gastrointestinal tract. Year of diagnosis was grouped into five categories: (1) 1980–1999, (2) 2000–2004, (3) 2005–2009, (4) 2010–2014, and (5) 2015–2019.

Economic status was classified using the record “median household income inflation-adjusted to 2019” as follows: (< USD 60,000) for the low-income group, (USD 60,000–74,999) for the middle-income group, and (≥ USD 75,000) for the high-income group. Geographic county areas were categorized into three groups based on population size: metropolitan areas with more than 1 million or less than 1 million residents and non-metropolitan areas recorded as “Rural-Urban Continuum.”

Tumor stage was classified as follows: (1) localized stage for tumors confined to the site of origin, (2) regional stage for tumors with direct extension or regional lymph node metastasis, and (3) distant stage for metastasis to distant sites or distant lymph nodes. Patient vital status and cause of death were obtained from the SEER database. Gastrointestinal stromal tumor-specific mortality (TSM) was defined as deaths directly caused by GIST, and tumor-specific survival (TSS) was measured as time from diagnosis to death or last follow-up.

### Statistical analysis

Categorical variables, namely age group, gender, ethnicity, primary tumor site, year of diagnosis, median household income, and geographic county area, were expressed as frequencies (percentages) and analyzed among different primary tumor sites using the Chi-square test. TSM across various age groups, genders, ethnicity, primary tumor sites, time periods, economic levels, and geographic categories was analyzed using Kaplan–Meier curves and compared using the Log-rank (Mantel-Cox) test. Additionally, TSS trends over different time periods were analyzed using the Log-rank test for trend. Five-year TSS rates across different groups were visualized using a heatmap.

Independent factors influencing TSM were identified through a multivariable Cox regression model, with results reported as adjusted hazard ratios (HR), 95% confidence intervals (CI), and p-values. A multivariable Cox regression model, that included age, gender, time periods, tumor stage, primary tumor site, and median household income, was also employed to assess associations between time periods and TSM across different subpopulations. In multivariable Cox regression model 1, patients in 1980–1999 served as the reference group. In model 2, patients in 2000–2004 were the reference group, excluding those from 1980 to 1999. In model 3, patients in 2005–2009 were the reference group, excluding those from 1980 to 2004.

All statistical analyses were conducted using SPSS software (version 26.0, IBM SPSS Inc., Chicago, IL, United States). A two-tailed p-value <0.05 was considered statistically significant. Graphs were generated using GraphPad Prism (version 8.0, GraphPad Software Inc., San Diego, CA, United States).

## Results

### Patient characteristics

Patient characteristics are summarized in Supplementary Table S1. Briefly, among the 4,127 GIST patients, the most common primary tumor site was the stomach (57.1%), followed by the small intestine (26.6%) and colorectum (4.7%), while 481 cases (11.7%) occurred outside the gastrointestinal tract. GISTs were more frequently observed in older adults (50.4%), followed by middle-aged adults (44.0%). The age distribution varied significantly across primary tumor sites, with GIST in the small intestine occurring at a younger mean age compared to other sites.

Tumors in different primary tumor sites showed similar gender distributions. GIST in the small intestine had a higher proportion of white patients. Patients with GIST in the stomach had the highest proportion of high-household income (54.9%), whereas those with tumors outside the gastrointestinal tract were more likely to belong to the low-household income group (16.7%). A higher proportion of patients with primary tumors in the stomach resided in metropolitan areas with populations greater than 1 million. In contrast, a higher proportion of GIST patients with tumors located outside the gastrointestinal tract (12.5%) or in the small intestine (12.0%) lived in non-metropolitan areas.

Tumor stage also differed by primary tumor site. GIST in the stomach had the highest proportion of localized lesions (66.1%). In contrast, small intestine GISTs were more frequently associated with regional lesions (21.2%), while tumors outside the gastrointestinal tract were more likely to be associated with distant lesions (42.0%).

### Risk factors for GIST mortality

**FIGURE 2:**
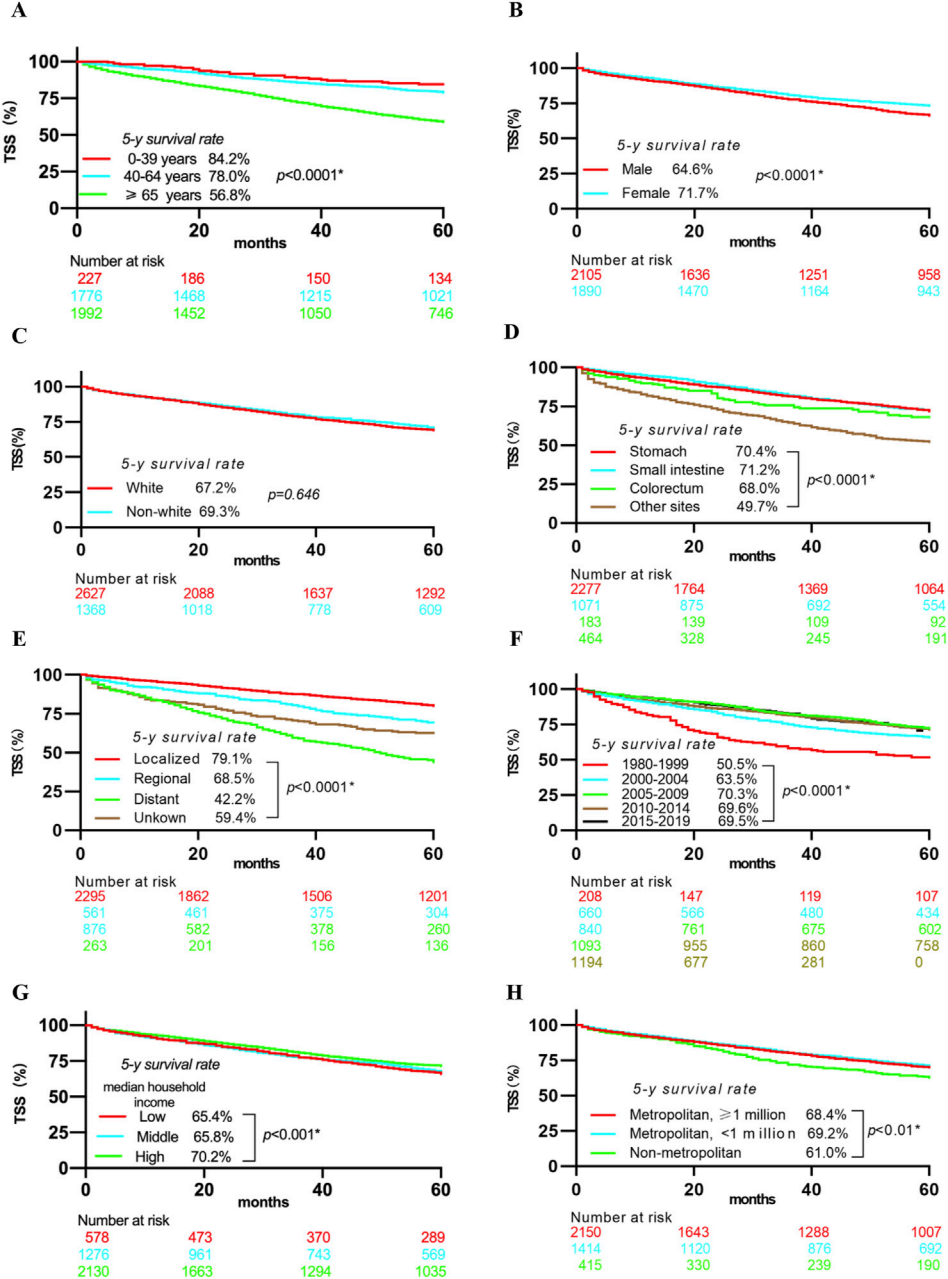
Kaplan-Meier curves showing 5-year TSS rates stratified by Age **(A)**, Gender **(B)**, Ethnicity **(C)**, Primary tumor site **(D)**, Disease stage **(E)**, year of diagnosis **(F)**, Median household income **(G)**, Geographic county area **(H)**. Notes: Abbreviations: TSS, Tumor-specific survival; * Statistically significant.

As shown in Figure 2, patients who were older (≥65 years), male, had tumors outside the gastrointestinal tract, had a distant-stage disease, were diagnosed between 1980 and 1999, belonged to low- or middle-income households, or lived in non-metropolitan areas had lower 5-year TSS rates.

**FIGURE 3:**
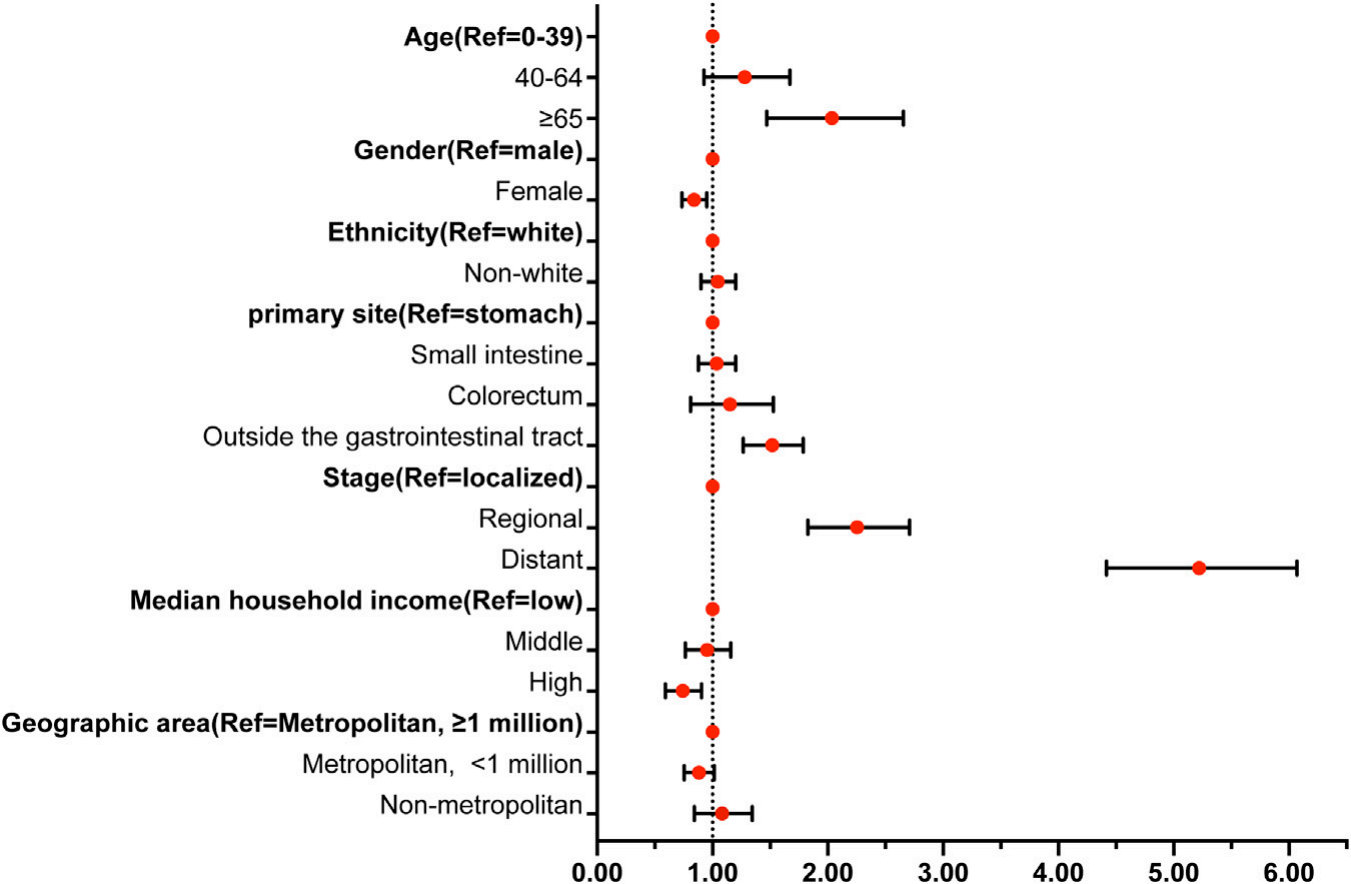
Forest map showing the independent risk factors of TSM. Notes: Abbreviations: TSM, Gastrointestinal stromal tumor-specific mortality; HR, hazard ratio; CI, confidence interval; * Statistically significant. Bold indicates a reference variable.

Multivariable analysis (Figure 3) identified several independent risk and protective factors for TSM. Older age (≥65 years) (HR = 1.977, 95% CI = 1.470–2.657), tumor locations outside the gastrointestinal tract (HR = 1.505, 95% CI = 1.267–1.786), regional lesions (HR = 2.225, 95% CI = 1.828–2.708), and distant lesions (HR = 5.177, 95% CI = 4.417–6.069) were independent risk factors for TSM (all p < 0.05). Conversely, being a woman (HR = 0.836, 95% CI = 0.735–0.951) and having a high household income (HR = 0.732, 95% CI = 0.592–0.905) were independent protective factors for TSM (p < 0.05).

To further evaluate the impact of time periods on TSM, these significant factors, which included age, gender, time periods, primary tumor location, disease stage, and median household income, were included in multivariable analysis models. Table 1 presents the adjusted HRs for TSM across different time periods after adjusting for age, gender, time periods, primary tumor location, disease stage, and median household income. When the time period 1980–1999 was set as the reference group, patients in 2000–2004 (HR = 0.662, 95% CI = 0.523–0.839), 2005–2009 (HR = 0.431, 95% CI = 0.339–0.549), 2010–2014 (HR = 0.437, 95% CI = 0.341–0.561), and 2015–2019 (HR = 0.365, 95% CI = 0.273–0.489) had a significantly lower risk of TSM than patients in 1980–1999 (p < 0.05). Similarly, when 2000–2004 was set as the reference group, patients diagnosed in 2005–2009 (HR = 0.661, 95% CI = 0.555–0.788), 2010–2014 (HR = 0.696, 95% CI = 0.578–0.838), and 2015–2019 (HR = 0.607, 95% CI = 0.476–0.773) also had a significantly lower risk of TSM than patients in 2000–2004 (p < 0.05). However, when 2005–2009 was used as the reference group, patients diagnosed in 2010–2014 (HR = 1.042, 95% CI = 0.863–1.258) and 2015–2019 (HR = 0.945, 95% CI = 0.734–1.216) did not exhibit significantly lower risk of TSM compared to those in 2005–2009 (p > 0.05).

**TABLE 1:** Trends in adjusted HR for TSM.

	Multivariable model 1	Multivariable model 2	Multivariable model 3
	Adjusted HR (95% CI)	Adjusted HR (95% CI)	Adjusted HR (95% CI)
Age			
<40 years old	reference	reference	reference
40–64 years old	1.82 (1.38–2.40)^a^	1.59 (1.17–2.16)^a^	1.70 (1.13–2.55)^a^
≥65 years old	4.58 (3.48–6.04)^a^	4.12 (3.05–5.56)^a^	4.71 (3.15–7.04)^a^
Gender			
Male patients	Reference	reference	reference
Female patients	0.77 (0.70–0.85)^a^	0.77 (0.69–0.85)^a^	0.80 (0.71–0.91)^a^
Time period			
1980–1999	reference	NA	NA
2000–2004	0.66 (0.52–0.84)^a^	reference	NA
2005–2009	0.43 (0.34–0.55)^a^	0.66 (0.55–0.79)^a^	reference
2010–2014	0.44 (0.34–0.56)^a^	0.70 (0.58–0.84)^a^	1.04 (0.86–1.26)
2015–2019	0.36 (0.27–0.49)^a^	0.61 (0.48–0.77)^a^	0.95 (0.73–1.22)
Median household incomes			
Low income	reference	reference	reference
Middle income	0.99 (0.86–1.15)	0.96 (0.83–1.12)	0.91 (0.76–1.08)
High income	0.85 (0.74–0.97)^a^	0.87 (0.76–1.01)	0.84 (0.71–0.99)^a^
Tumor Stage			
Localized	reference	reference	reference
Regional	1.60 (1.39–1.84)^a^	1.55 (1.33–1.80)^a^	1.66 (1.37–2.00)^a^
Distant	3.21 (2.86–3.61)^a^	3.09 (2.74–3.49)^a^	3.25 (2.81–3.75)^a^
Primary tumor site			
Stomach	reference	reference	reference
Small Intestine	0.93 (0.83–1.05)	0.97 (0.85–1.09)	0.95 (0.81–1.10)
Colorectum	1.05 (0.83–1.32)	1.00 (0.78–1.29)	1.04 (0.76–1.42)
Outside the Gastrointestinal Tract	1.28 (1.12–1.47)^a^	1.41 (1.22–1.62)^a^	1.54 (1.30–1.82)^a^

Notes: In multivariable model 1, the period 1980–1999 was used as the reference year. Adjusted HRs, were calculated after controlling for patient age, gender, tumor stage, time periods, primary tumor site, and median household income. In multivariable model 2, the period 2000–2004 was used as the reference year, with adjusted HRs, calculated using the same control variables. In multivariable model 3, the period 2005–2009 was used as the reference year, and adjusted HRs, were calculated similarly.

^a^ Indicates statistical significance.

Abbreviations: TSM, gastrointestinal stromal tumor-specific mortality; HR, hazard ratio; CI, confidence interval.

### Changes in 5-year GIST survival rates

**FIGURE 4:**
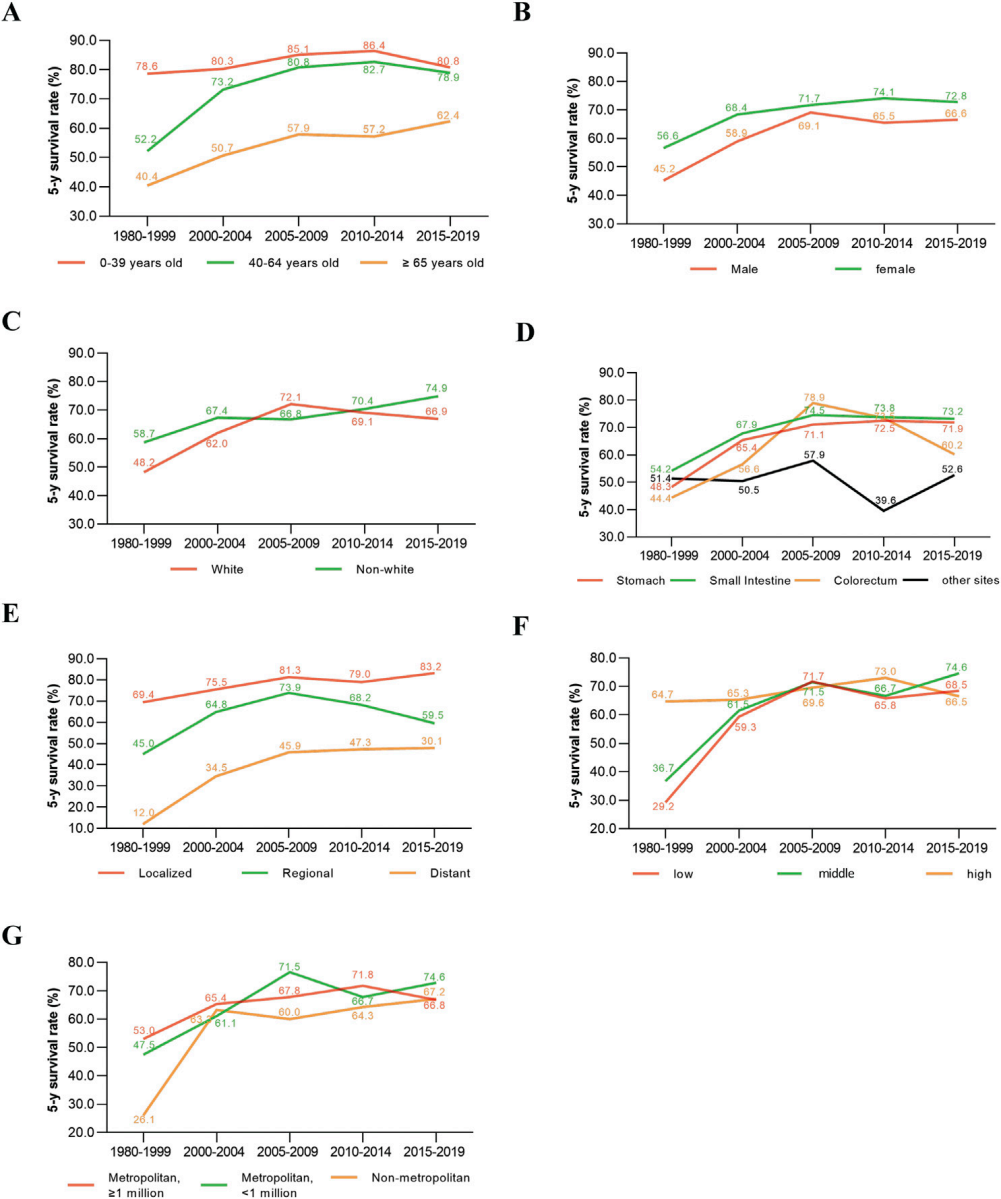
Change in 5-year survival rates of GIST patients in different time periods stratified by Age **(A)**, Gender **(B)**, Ethnicity **(C)**, Primary tumor site **(D)**, Disease Stage **(E)**, median household income **(F)**, Geographic county area **(G)**.

Compared to patients diagnosed in 1980–1999, those diagnosed in 2015–2019 showed significant improvements in 5-year TSS rates across various subgroups. Middle-aged patients (40–64 years old) experienced a 26.7% improvement, while older patients (≥65 years old) improved by 22.0%. In contrast, young patients (0–39 years old) showed only a modest 2.2% increase in their 5-year TSS rate (Figure 4A). Male and female patients improved their 5-year TSS rate by 21.4% and 16.2%, respectively (Figure 4B). Similarly, white and non-white patients experienced improvements of 18.7% and 16.2%, respectively (Figure 4C). For primary tumor locations, patients with GIST in the stomach, small intestine, and colorectum had 5-year TSS rate improvements of 23.6%, 19.0%, and 15.8%, respectively. However, patients with primary tumors outside the gastrointestinal tract showed only a 1.2% increase in their 5-year TSS rate from 1980 to 1999 to 2015–2019 (Figure 4D). Patients with distant lesions had the most significant improvement, with their 5-year TSS rate increasing from 12.0% in 1980–1999 to 30.1% in 2015–2019. In comparison, patients with localized and regional lesions improved their 5-year TSS rates by 13.8% and 14.5%, respectively (Figure 4E). Patients with low- and middle-household incomes improved their 5-year TSS rates by 39.3% and 37.9%, respectively, while patients with high household incomes showed little change in their 5-year TSS rate over the same period (Figure 4F). Regarding geographic areas, patients living in metropolitan areas with populations greater than 1 million, metropolitan areas with populations less than 1 million, and non-metropolitan areas improved their 5-year TSS rates by 13.8%, 27.1%, and 41.1%, respectively, from 1980 to 1999 to 2015–2019 (Figure 4G).

### Associations between time periods and TSM in specific primary tumor sites and at specific stages of the disease

As shown in Supplementary Figure S1, GISTs with primary tumor sites in the stomach, small intestine, and outside the gastrointestinal tract showed significant increases in 5-year TSS rates (Supplementary Figures S1A, B, D, all p < 0.05). However, GISTs with primary tumor sites in the colorectum did not show statistically significant differences in 5-year TSS rates across different time periods (Supplementary Figure S1C). Supplementary Figure S2 shows that patients with localized or distant lesions significantly improved their 5-year TSS rates over time (both p < 0.05). In contrast, those with regional lesions showed no significant survival changes across different time periods (p > 0.05). A heatmap depicting survival changes in different time periods, primary tumor sites, and disease stages is shown in Supplementary Figure S3.

GIST patients had a distinct survival trend in different primary tumor sites and tumor stages. Patients with primary tumor locations in the stomach exhibited consistently lower risk of TSM in 2005–2019 than patients in 1980–1999 and 2000–2004 (all p < 0.05). Patients with primary tumor locations in the small intestine exhibited a lower risk of TSM in 2000–2019 than patients in 1980–1999 (all p < 0.05). However, GISTs in the colorectum and outside the gastrointestinal tract showed a similar risk of TSM across all time periods (all p > 0.05) (Table 2). Patients with distant-stage tumors diagnosed in 2000–2019 showed consistently lower risk of TSM than patients in 1980–1999 (all p < 0.05). Patients with localized or distant lesions diagnosed in 2005–2019 also demonstrated consistently lower risk of TSM compared to those diagnosed in 2000–2004 (all p < 0.05). However, patients diagnosed in 2010–2014 and 2015–2019 showed no significant differences in TSM risk compared to those diagnosed in 2005–2009, regardless of disease stage (all p > 0.05) (Table 3).

**TABLE 2:** Trends in adjusted HR for primary site-specific TSM.

Decade	1980–1999	2000–2004	2005–2009	2010–2014	2015–2019
Primary tumor site		Adjusted HR (95% CI)	Adjusted HR (95% CI)	Adjusted HR (95% CI)	Adjusted HR (95% CI)
Multivariable model 1					
Stomach	1.00	0.438 (0.302–0.634)^a^	0.258 (0.177–0.374)^a^	0.228 (0.156–0.333)^a^	0.180 (0.117–0.278)^a^
Small intestine	1.00	0.623 (0.406–0.956)^a^	0.408 (0.261–0.637)^a^	0.416 (0.264–0.657)^a^	0.396 (0.224–0.702)^a^
Colorectum	1.00	0.720 (0.286–1.817)	0.200 (0.065–0.618)^a^	0.315 (0.104–0.956)^a^	0.396 (0.122–1.278)
Outside the gastrointestinal tract	1.00	1.049 (0.611–1.800)	0.848 (0.495–1.453)	1.283 (0.741–2.223)	0.950 (0.504–1.792)
Multivariable model 2					
Stomach	-	1.00	0.598 (0.460–0.777)^a^	0.529 (0.399–0.701)^a^	0.431 (0.302–0.615)^a^
Small intestine	-	1.00	0.668 (0.479–0.931)^a^	0.684 (0.480–0.975)^a^	0.698 (0.428–1.138)
Colorectum	-	1.00	0.312 (0.108–0.896)^a^	0.512 (0.182–1.437)	0.724 (0.227–2.311)
Outside the gastrointestinal tract	-	1.00	0.808 (0.551–1.185)	1.277 (0.867–1.880)	0.988 (0.599–1.629)
Multivariable model 3					
Stomach	-	-	1.00	0.872 (0.663–1.146)	0.746 (0.520–1.069)
Small intestine	-	-	1.00	1.014 (0.762–1.351)	0.874 (0.580–1.316)
Colorectum	-	-	1.00	1.772 (0.477–6.583)	3.699 (0.765–17.879)
Outside the gastrointestinal tract	-	-	1.00	1.579 (1.063–2.346)^a^	1.220 (0.720–2.067)

Notes: In multivariable model 1, the period 1980–1999 was used as the reference year. Adjusted HRs, were calculated after controlling for patient age, gender, tumor stage, time periods, and median household income. In multivariable model 2, the period 2000–2004 was used as the reference year, with adjusted HRs, calculated using the same control variables. In multivariable model 3, the period 2005–2009 was used as the reference year, and adjusted HRs, were calculated similarly.

^a^ Indicates statistical significance.

Abbreviations: TSM, gastrointestinal stromal tumor-specific mortality; HR, hazard ratio; CI, confidence interval.

**TABLE 3:** Trends in adjusted HR for disease stage-specific TSM.

Time period	1980–1999	2000–2004	2005–2009	2010–2014	2015–2019
Stage		Adjusted HR (95% CI)	Adjusted HR (95% CI)	Adjusted HR (95% CI)	Adjusted HR (95% CI)
Multivariable model 1					
Localized	1.00	0.745 (0.494–1.126)	0.452 (0.291–0.700)^a^	0.380 (0.241–0.597)^a^	0.414 (0.241–0.709)^a^
Regional	1.00	0.578 (0.321–1.039)	0.353 (0.192–0.650)^a^	0.443 (0.293–0.821)^a^	0.217 (0.087–0.546)^a^
Distant	1.00	0.431 (0.294–0.633)^a^	0.299 (0.206–0.436)^a^	0.287 (0.196–0.421)^a^	0.232 (0.151–0.355)^a^
Multivariable model 2					
Localized	-	1.00	0.620 (0.445–0.863)^a^	0.534 (0.375–0.760)^a^	0.599 (0.376–0.954)^a^
Regional	-	1.00	0.631 (0.422–0.942)^a^	0.798 (0.507–1.256)	0.438 (0.193–0.996)^a^
Distant	-	1.00	0.697 (0.529–0.917)^a^	0.676 (0.508–0.898)^a^	0.566 (0.401–0.798)^a^
Multivariable model 3					
Localized	-	-	1.00	0.836 (0.575–1.216)	0.960 (0.584–1.578)
Regional	-	-	1.00	1.253 (0.774–2.028)	0.744 (0.317–1.745)
Distant	-	-	1.00	0.979 (0.743–1.290)	0.866 (0.610–1.229)

Notes: In multivariable model 1, the period 1980–1999 was used as the reference year. Adjusted HRs, were calculated after controlling for patients’ age, gender, time periods, primary tumor site, and median household income. In multivariable model 2, the period 2000–2004 was used as the reference year, with adjusted HRs, calculated using the same control variables. In multivariable model 3, the period 2005–2009 was used as the reference year, and adjusted HRs, were calculated similarly.

^a^ Indicates statistical significance.

Abbreviations: TSM, gastrointestinal stromal tumor-specific mortality; HR, hazard ratio; CI, confidence interval.

## Discussion

GIST, a rare soft tissue sarcoma, has shown an increasing prevalence worldwide over the past decades [10–12]. With advances in diagnostic techniques and the development of more precise anticancer therapies, the majority of cancer patients have experienced significant improvements in median overall survival [13–15]. Ulrich Guller [8] has previously reported survival trends in GIST patients, showing increased overall and cancer-specific survival from 1998 to 2008. However, unlike high-incidence cancers, few studies have focused on survival trends in GIST patients since then, particularly to identify which subgroups have benefited from updated diagnostic and therapeutic approaches.

This study utilized the most recent data to explore survival outcomes and trends in GIST patients over the past 40 years. The 5-year survival rates for GIST patients were 50.5%, 63.5%, 70.3%, 69.6%, and 69.5% for the periods 1980–1999, 2000–2004, 2005–2009, 2010–2014, and 2015–2019, respectively. The trends showed significant improvement in 5-year TSS rates from 2000 to 2009, followed by a stabilization from 2010 to 2019. Tumor stage, older age, and primary tumor location emerged as the three most significant factors influencing patient survival outcomes, emphasizing the need for more targeted measures to improve the prognosis of GIST patients with regional or distant-stage disease, of advanced age, and with primary tumors outside the gastrointestinal tract.

The tumor stage is a critical factor affecting GIST prognosis. The majority of localized GIST patients can be cured with surgery, and their survival trends remain optimistic. However, in real-world settings, 5-year survival trends for inoperable GIST patients have not improved since 2005. Significant progress has been made in treating locally advanced and metastatic GISTs. In 1998, Hirota [16] identified c-KIT gene mutations in GIST, a discovery that led to substantial advances in the understanding of the disease. Approximately 80% of GIST patients harbor c-KIT mutations, while 10%–15% have PDGFRA mutations [17, 18]. This discovery enabled the development of imatinib, a tyrosine kinase inhibitor (TKI), which became the first-line treatment for GIST and significantly improved survival outcomes [19].

The findings in this study demonstrate substantial survival improvements for patients diagnosed between 2005 and 2019 compared to those diagnosed in earlier periods. However, patients who develop resistance to TKIs, either initially or during treatment, face limited efficacy with second or third-line therapies and are prone to further drug resistance. This likely explains why survival rates have plateaued from 2010 to 2019. Despite these challenges, ongoing research has focused on addressing TKI resistance by exploring new KIT signaling pathways and developing targeted drugs against KIT mutations [20, 21]. For example, ripretinib has been established as the standard fourth-line treatment for advanced GIST, extending survival by 15.1 months in patients who have developed resistance to imatinib, sunitinib, and regorafenib [22]. In addition to TKIs, cytoreductive surgery has been investigated as a potential option for advanced GIST. Studies suggest that cytoreductive surgery may benefit patients with localized progression or radiographic responses to TKI treatment, improving progression-free survival (PFS) and overall survival (OS) [23–25]. Furthermore, ongoing clinical trials are exploring immunotherapy agents for GIST, offering hope for future advances [26, 27].

The primary tumor location has a significant impact on GIST survival outcomes. The stomach was found to be the most common primary tumor site, accounting for 57.1% of cases. The 5-year TSS rates for stomach GIST patients were 48.8%, 65.8%, 72.2%, 73.6%, and 73.1% for the periods 1980–1999, 2000–2004, 2005–2009, 2010–2014, and 2015–2019, respectively. The second most common site was the small intestine (26.6% of cases), with corresponding 5-year survival rates of 48.3%, 65.4%, 71.1%, 72.5%, and 71.9%. While previous studies have suggested differences in clinical outcomes between stomach and small intestine GISTs [28, 29], our findings indicate that their 5-year survival rates and trends are similar, showing improvement before 2009 and stabilization after 2010. This could be attributed to the patient selection criteria and the time periods of enrollment in the study.

In contrast, GISTs in the colorectum exhibited fluctuating trends, with the 5-year survival rate increasing from 48.3% in 1980–1999 to 78.9% in 2005–2009, followed by a decline to 60.2% in 2015–2019. Multivariable analysis did not identify colorectal GIST as an independent risk factor for TSM, suggesting that variations in patient age and tumor stage across different periods may have influenced these trends. For GISTs occurring outside the gastrointestinal tract, survival rates remained unchanged from 1980 to 1999 to 2005–2019, indicating poorer outcomes. Our study also revealed that the risk of TSM increased for GISTs outside the gastrointestinal tract in the period 2010–2014. GISTs located outside the gastrointestinal tract represent a unique entity with distinct characteristics and outcomes from GISTs that occur in the gastrointestinal tract [8, 30]. However, as the incidence of GISTs located outside the gastrointestinal tract is relatively low, and there is an absence of specific treatment guidelines and large randomized controlled trials for these patients, treatment strategies are typically based on general GIST protocols. This approach may lead to undertreatment or overtreatment, and there is a need to improve precision therapy for this specific patient population. Studies have shown that non-gastric GISTs are associated with worse recurrence-free survival and are considered independent adverse prognostic factors following surgery [31, 32]. This may partially explain the poor outcomes for non-gastrointestinal GISTs.

This study has several limitations. First, the SEER database lacks genetic data, preventing analysis of survival trends in patients with or without c-KIT mutations. Additionally, limited treatment information and the absence of key clinical factors such as mitotic index and risk stratification restricted our ability to fully explore prognostic factors. Despite these limitations, our study provides valuable insights into the survival trends of GIST patients in real-world settings, helping to identify precise target populations for future interventions aimed at improving survival outcomes.

## Conclusion

In summary, the survival of GIST patients significantly improved between 2000 and 2009 but showed no substantial improvement from 2010 to 2019, with the turning point for lower risk of TSM being 2005. Innovative strategies are urgently needed to improve the outcomes for GIST patients, particularly for older patients and those with tumors originating outside the gastrointestinal tract.

## Data Availability

Publicly available datasets were analyzed in this study. This data can be found here: The datasets generated and/or analysed during the current study are available in the Surveillance, Epidemiology, and End Results Program repository, https://seer.cancer.gov/data/.

## Footnotes

1
https://seer.cancer.gov/

## References

[1] Blay JY Kang YK Nishida T von Mehren M. Gastrointestinal stromal tumours. Nat Rev Dis Primers (2021) 7(1):22. 10.1038/s41572-021-00254-5 33737510

[2] Wada R Arai H Kure S Peng WX Naito Z. Wild type GIST: clinicopathological features and clinical practice. Pathol Int (2016) 66(8):431–7. 10.1111/pin.12431 27427238

[3] Kelly CM Gutierrez Sainz L Chi P. The management of metastatic GIST: current standard and investigational therapeutics. J Hematol Oncol (2021) 14(1):2. 10.1186/s13045-020-01026-6 33402214 PMC7786896

[4] Lu T Yang X Huang Y Zhao M Li M Ma K Trends in the incidence, treatment, and survival of patients with lung cancer in the last four decades. Cancer Manag Res (2019) 11:943–53. 10.2147/CMAR.S187317 30718965 PMC6345192

[5] Han J Wang B Liu W Wang S Chen R Chen M Declining disease burden of HCC in the United States, 1992-2017: a population-based analysis. Hepatology (2022) 76(3):576–88. 10.1002/hep.32355 35073427

[6] Everatt R Gudaviciene D. An analysis of time trends in breast and prostate cancer mortality rates in Lithuania, 1986-2020. BMC Public Health (2022) 22(1):1812. 10.1186/s12889-022-14207-4 36151551 PMC9508783

[7] Gold JS Gonen M Gutierrez A Broto JM García-del-Muro X Smyrk TC Development and validation of a prognostic nomogram for recurrence-free survival after complete surgical resection of localised primary gastrointestinal stromal tumour: a retrospective analysis. Lancet Oncol (2009) 10(11):1045–52. 10.1016/S1470-2045(09)70242-6 19793678 PMC3175638

[8] Guller U Tarantino I Cerny T Schmied BM Warschkow R. Population-based SEER trend analysis of overall and cancer-specific survival in 5138 patients with gastrointestinal stromal tumor. BMC Cancer (2015) 15:557. 10.1186/s12885-015-1554-9 26223313 PMC4518595

[9] Khan J Ullah A Waheed A Karki N Adhikari N Vemavarapu L Gastrointestinal stromal tumors (GIST): a population-based study using the SEER database, including management and recent advances in targeted therapy. Cancers (Basel) (2022) 14(15):3689. 10.3390/cancers14153689 35954353 PMC9367571

[10] Patel N Benipal B. Incidence of gastrointestinal stromal tumors in the United States from 2001-2015: a United States cancer statistics analysis of 50 states. Cureus (2019) 11(2):e4120. 10.7759/cureus.4120 31037234 PMC6478492

[11] Mantese G. Gastrointestinal stromal tumor: epidemiology, diagnosis, and treatment. Curr Opin Gastroenterol (2019) 35(6):555–9. 10.1097/MOG.0000000000000584 31577561

[12] Cho MY Sohn JH Kim JM Kim KM Park YS Kim WH Current trends in the epidemiological and pathological characteristics of gastrointestinal stromal tumors in Korea, 2003-2004. J Korean Med Sci (2010) 25(6):853–62. 10.3346/jkms.2010.25.6.853 20514305 PMC2877229

[13] Allemani C Matsuda T Di Carlo V Harewood R Matz M Nikšić M Global surveillance of trends in cancer survival 2000-14 (CONCORD-3): analysis of individual records for 37 513 025 patients diagnosed with one of 18 cancers from 322 population-based registries in 71 countries. Lancet (2018) 391(10125):1023–75. 10.1016/S0140-6736(17)33326-3 29395269 PMC5879496

[14] Allemani C Weir HK Carreira H Harewood R Spika D Wang X Global surveillance of cancer survival 1995-2009: analysis of individual data for 25,676,887 patients from 279 population-based registries in 67 countries (CONCORD-2). Lancet (2015) 385(9972):977–1010. 10.1016/S0140-6736(14)62038-9 25467588 PMC4588097

[15] Coleman MP Quaresma M Berrino F Lutz J Angelis RD Capocaccia R Cancer survival in five continents: a worldwide population-based study (CONCORD). Lancet Oncol (2008) 9(8):730–56. 10.1016/S1470-2045(08)70179-7 18639491

[16] Hirota S Isozaki K Moriyama Y Hashimoto K Nishida T Ishiguro S Gain-of-function mutations of c-kit in human gastrointestinal stromal tumors. Science (1998) 279(5350):577–80. 10.1126/science.279.5350.577 9438854

[17] Mohammadi M Gelderblom H. Systemic therapy of advanced/metastatic gastrointestinal stromal tumors: an update on progress beyond imatinib, sunitinib, and regorafenib. Expert Opin Investig Drugs (2021) 30(2):143–52. 10.1080/13543784.2021.1857363 33252274

[18] Wang MX Devine C Segaran N Ganeshan D. Current update on molecular cytogenetics, diagnosis and management of gastrointestinal stromal tumors. World J Gastroenterol (2021) 27(41):7125–33. 10.3748/wjg.v27.i41.7125 34887632 PMC8613640

[19] Klug LR Khosroyani HM Kent JD Heinrich MC. New treatment strategies for advanced-stage gastrointestinal stromal tumours. Nat Rev Clin Oncol (2022) 19(5):328–41. 10.1038/s41571-022-00606-4 35217782 PMC11488293

[20] Martin-Broto J Moura DS. New drugs in gastrointestinal stromal tumors. Curr Opin Oncol (2020) 32(4):314–20. 10.1097/CCO.0000000000000642 32541319

[21] Serrano C. New treatments in advanced gastrointestinal stromal tumor. Curr Opin Oncol (2021) 33(4):323–8. 10.1097/CCO.0000000000000745 33867479

[22] Blay JY Serrano C Heinrich MC Zalcberg J Bauer S Gelderblom H Ripretinib in patients with advanced gastrointestinal stromal tumours (INVICTUS): a double-blind, randomised, placebo-controlled, phase 3 trial. Lancet Oncol (2020) 21(7):923–34. 10.1016/S1470-2045(20)30168-6 32511981 PMC8383051

[23] Fernandez JA Alconchel F Gomez B Martinez J Ramirez P. Unresectable GIST liver metastases and liver transplantation: a review and theoretical basis for a new indication. Int J Surg (2021) 94:106126. 10.1016/j.ijsu.2021.106126 34592432

[24] Keung EZ Fairweather M Raut CP. The role of surgery in metastatic gastrointestinal stromal tumors. Curr Treat Options Oncol (2016) 17(2):8. 10.1007/s11864-015-0384-y 26820287

[25] Yonkus JA Alva-Ruiz R Grotz TE. Surgical management of metastatic gastrointestinal stromal tumors. Curr Treat Options Oncol (2021) 22(5):37. 10.1007/s11864-021-00837-0 33743084

[26] Fairweather M Balachandran VP Li GZ Bertagnolli MM Antonescu C Tap W Cytoreductive surgery for metastatic gastrointestinal stromal tumors treated with tyrosine kinase inhibitors: a 2-institutional analysis. Ann Surg (2018) 268(2):296–302. 10.1097/SLA.0000000000002281 28448384 PMC6203295

[27] Fairweather M Cavnar MJ Li GZ Bertagnolli MM DeMatteo RP Raut CP. Prediction of morbidity following cytoreductive surgery for metastatic gastrointestinal stromal tumour in patients on tyrosine kinase inhibitor therapy. Br J Surg (2018) 105(6):743–50. 10.1002/bjs.10774 29579329 PMC7938825

[28] Fero KE Coe TM Fanta PT Tang CM Murphy JD Sicklick JK. Surgical management of adolescents and young adults with gastrointestinal stromal tumors: a US population-based analysis. JAMA Surg (2017) 152(5):443–51. 10.1001/jamasurg.2016.5047 28114506 PMC5560852

[29] Miettinen M Lasota J. Gastrointestinal stromal tumors. Gastroenterol Clin North Am (2013) 42(2):399–415. 10.1016/j.gtc.2013.01.001 23639648 PMC3644178

[30] Liu L Feng Y Ye Y Wang Z Xu X. Survival analysis of extragastrointestinal stromal tumors based on the SEER database: a population-based study. Surg Endosc (2023) 37(11):8498–510. 10.1007/s00464-023-10433-y 37770606

[31] Joensuu H Vehtari A Riihimaki J Nishida T Steigen SE Brabec P Risk of recurrence of gastrointestinal stromal tumour after surgery: an analysis of pooled population-based cohorts. Lancet Oncol (2012) 13(3):265–74. 10.1016/S1470-2045(11)70299-6 22153892

[32] Zhang H Liu Q. Prognostic indicators for gastrointestinal stromal tumors: a review. Transl Oncol (2020) 13(10):100812. 10.1016/j.tranon.2020.100812 32619820 PMC7327422

