# Supplementary material for: Survival trends of gastrointestinal stromal tumor in real-world settings: a population-based retrospective study
Source: Pathol Oncol Res. 2025 Mar 4;31:1611896. doi: 10.3389/pore.2025.1611896 (PMC11913614; doi:10.3389/pore.2025.1611896)
Supplement: Supplementary file 1 [file Presentation1.zip › Supplementary Material/table S1.docx]

**Table S1** Demographic and neoplastic characteristics of the patients

|  | **All**  **N=4127** | **Stomach**  **N=2356** | **Small Intestine**  **N=1096** | **Colorectum**  **N=194** | **Outside the Gastrointestinal Tract**  **N=481** |
| --- | --- | --- | --- | --- | --- |
| **Age（years）*** |  |  |  |  |  |
| < 40 year old | 231(5.6%) | 123(5.2%) | 81(7.4%) | 10(5.1%) | 17(3.5%) |
| 40-64 years old | 1815(44.0%) | 949(40.3%) | 560(38.8%) | 94(48.5%) | 212(44.1%) |
| ≥65 years old | 2081(50.4%) | 1284(54.5%) | 455(51.1%) | 90(46.4%) | 252(52.4%) |
| **Gender** |  |  |  |  |  |
| Male | 2178(52.8%) | 1206(50.9%) | 616(57.1%) | 105(54.1%) | 251(52.2%) |
| Female | 1949(47.2%) | 1150(49.1%) | 480(42.9%) | 89(45.9%) | 230(47.8%) |
| **Ethnicity*** |  |  |  |  |  |
| White | 2718(65.9%) | 1419(60.2%) | 832(75.9%) | 117(60.3%) | 350(72.8%) |
| Non-white | 1409(34.1%) | 937(39.8%) | 264(24.1%) | 77(39.7%) | 131(27.2%) |
| **Year of diagnosis*** |  |  |  |  |  |
| Before 2000 | 213(5.2%) | 86(3.7%) | 72(6.6%) | 19(9.8%) | 36(7.5%) |
| 2000-2004 | 685(16.6%) | 328(13.9%) | 219(20.0%) | 35(18.1%) | 103(21.4%) |
| 2005-2009 | 863(20.9%) | 471(20.0%) | 224(20.4%) | 40(20.6%) | 128(26.6%) |
| 2010-2014 | 1122(27.2%) | 679(28.8%) | 283(25.8%) | 52(26.8%) | 108(22.5%) |
| 2015-2019 | 1244(30.1%) | 792(33.6%) | 298(27.2%) | 48(24.7%) | 106(22.0%) |
| **Median household incomes*** |  |  |  |  |  |
| Low-income | 598(14.5%) | 337(14.3%) | 155(23.3%) | 26(13.4%) | 80(16.6%) |
| Middle- income | 1322(32.0%) | 719(30.5%) | 359(32.8%) | 73(37.6%) | 172(35.8%) |
| High-income | 2195(53.2%) | 1294(54.9%) | 580(52.9%) | 94(48.5%) | 227(47.2%) |
| **Geographic areas*** |  |  |  |  |  |
| Metropolitan with over million | 2222(53.8%) | 1304(55.3%) | 560(51.1%) | 111(57.2%) | 247(51.3%) |
| Metropolitan with less than 1 million | 1459(35.4%) | 817(34.7%) | 401(36.6%) | 69(35.6%) | 172(35.8%) |
| Non-metropolitan | 430(10.4%) | 225(9.6%) | 132(12.0%) | 13(6.7%) | 60(12.5%) |
| **Tumor Stage*** |  |  |  |  |  |
| Localized | 2351(57.0%) | 1558(66.1%) | 592(54.0%) | 113(58.2%) | 88(18.3%) |
| Regional | 573(13.9%) | 252(10.7%) | 189(17.2%) | 42(21.6%) | 90(18.7%) |
| Distant | 918(22.2%) | 419(17.8%) | 269(24.5%) | 28(14.4%) | 202(42.0%) |

**Notes:** * Statistically significant, p<0.05.
