# Supplementary figures and images for: Survival trends of gastrointestinal stromal tumor in real-world settings: a population-based retrospective study
Source: Pathol Oncol Res. 2025 Mar 4;31:1611896. doi: 10.3389/pore.2025.1611896 (PMC11913614; doi:10.3389/pore.2025.1611896)

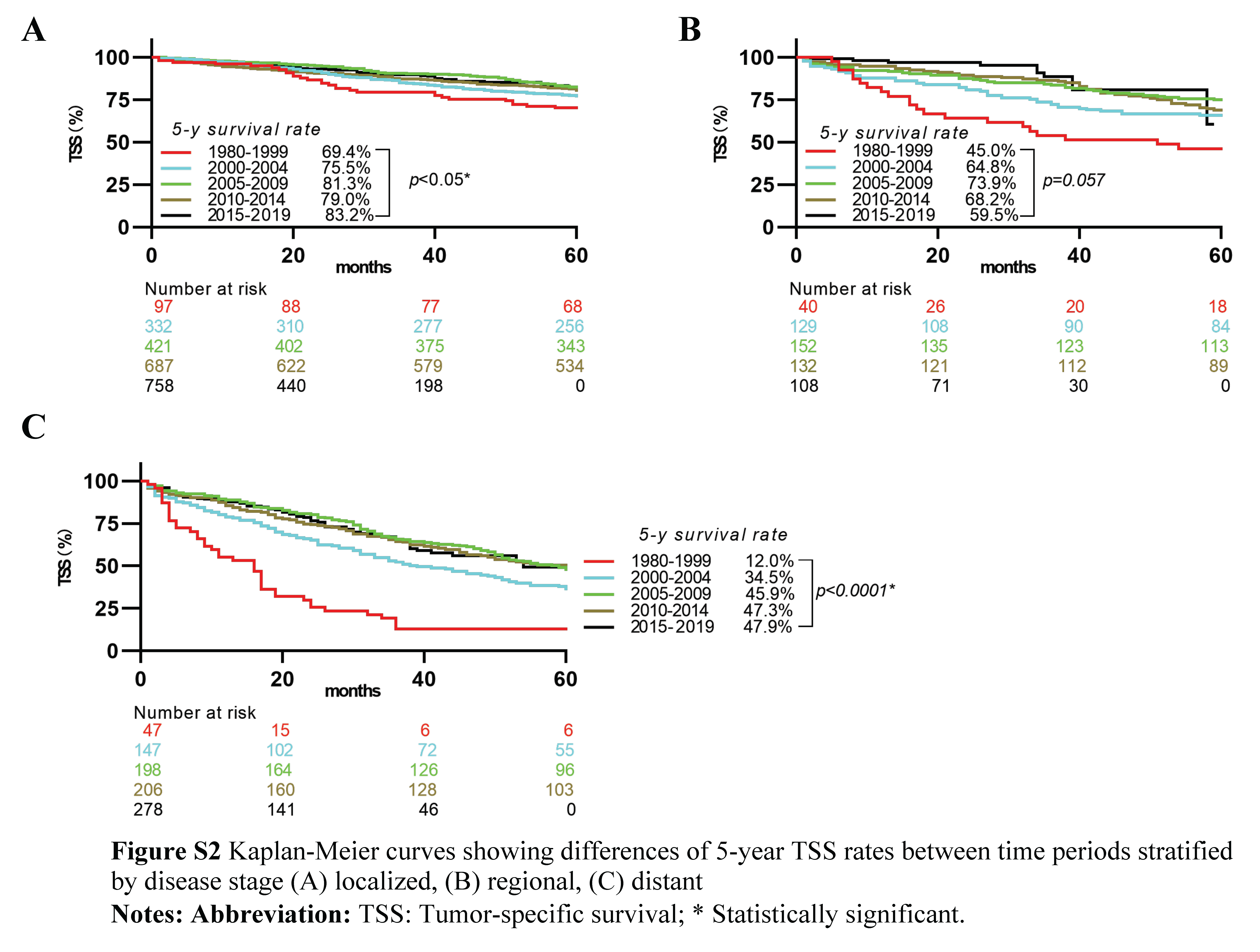

Supplement: Supplementary file 1 [file Presentation1.zip › Supplementary Material/figure S2.tiff]

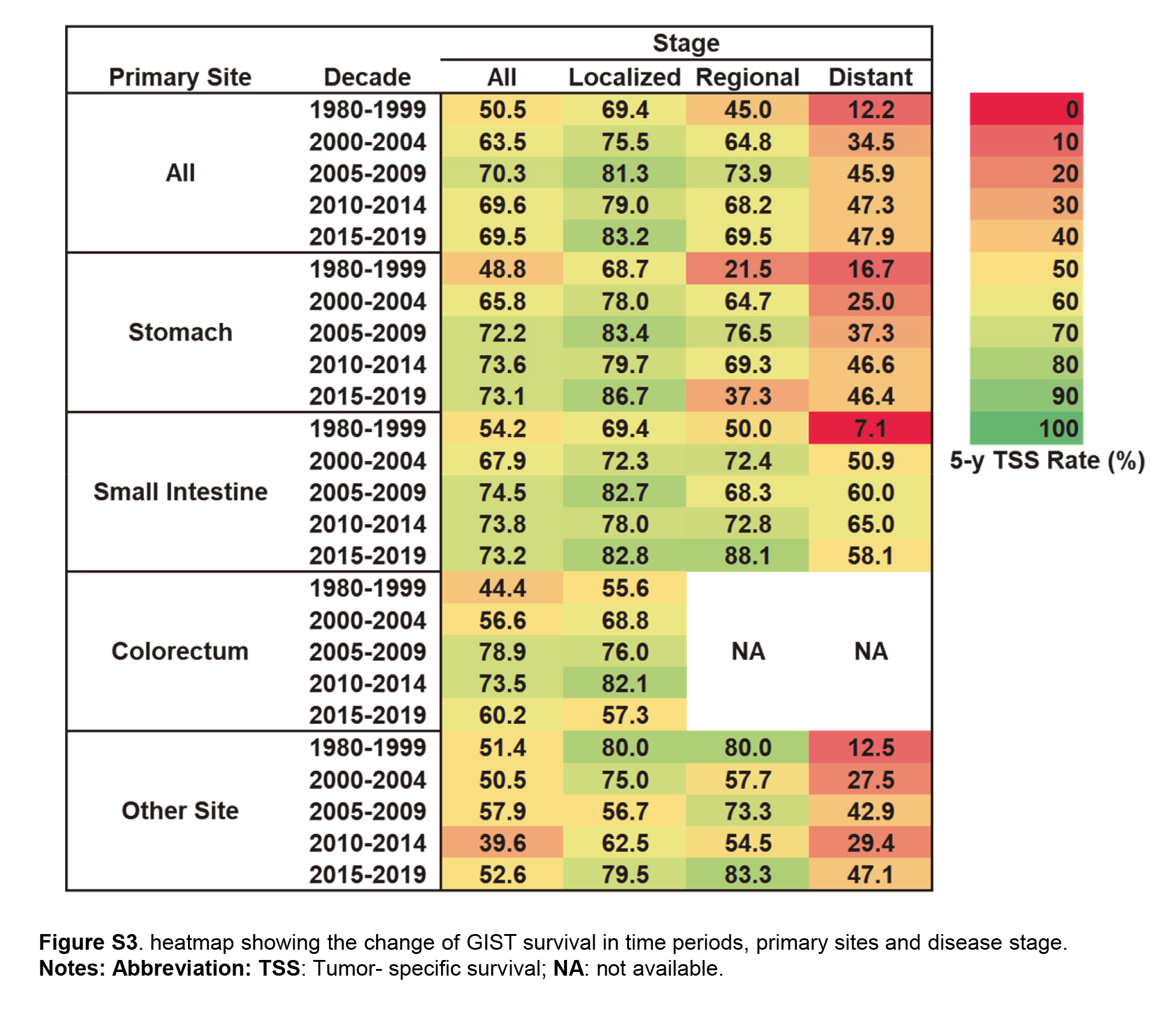

Supplement: Supplementary file 1 [file Presentation1.zip › Supplementary Material/Figure S3.tif]

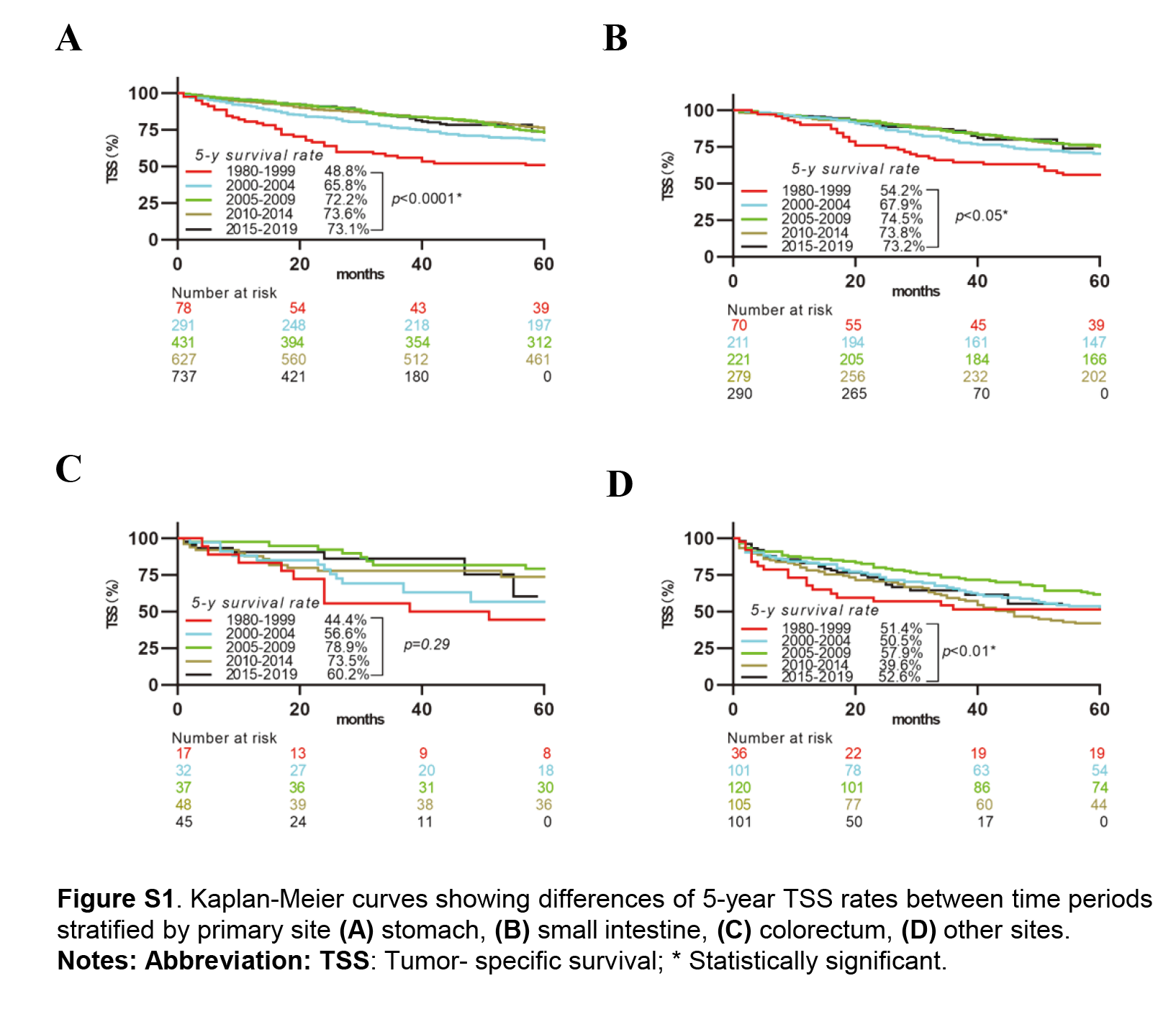

Supplement: Supplementary file 1 [file Presentation1.zip › Supplementary Material/Figure S1.tif]
